# Lower Blood Glucose and Variability Are Associated with Earlier Recovery from Renal Injury Caused by Episodic Urinary Tract Infection in Advanced Type 2 Diabetic Chronic Kidney Disease

**DOI:** 10.1371/journal.pone.0108531

**Published:** 2014-09-26

**Authors:** Ping-Fang Chiu, Chia-Lin Wu, Ching-Hui Huang, Hung-Hsiang Liou, Chirn-Bin Chang, Horng-Rong Chang, Chia-Chu Chang

**Affiliations:** 1 Institute of Medicine, Chung Shan Medical University, Taichung, Taiwan; 2 School of Medicine, Chung Shan Medical University, Taichung, Taiwan; 3 Nephrology Division, Department of Internal Medicine, Changhua Christian Hospital, Changhua, Taiwan; 4 Division of Cardiology, Department of Internal Medicine, Changhua Christian Hospital, Changhua, Taiwan; 5 Division of Nephrology, Department of Internal Medicine, Chung Shan Medical University Hospital, Taichung, Taiwan; 6 Ph.D. Program for Aging, College of Medicine, China Medical University, Taichung, Taiwan; San Raffaele Hospital, Italy

## Abstract

**Purpose:**

In our previous study, type 2 diabetic chronic kidney disease (CKD) patients with glomerular filtration rates of <30 mL/min upon hospitalization for urinary tract infection (UTI) were at a risk for acute kidney injury. This study aimed to clarify the effect of glucose and its variability on renal outcomes during admission for the treatment of UTI.

**Materials and Methods:**

Based on the date of renal recovery (RIFLE criteria: acute kidney injury occurred within 1–7 days and was sustained over 1 day), we divided these patients into early- (≤9 days, Group A) and late-recovery (>9 days, Group B) groups. The differences in the continuous and categorical variables of the two groups were assessed separately. The mean glucose levels and their variability (using the standard deviation and the coefficient of standard deviation) were compared at the fasting, midday pre-meal, evening pre-meal, and evening post-meal time points during hospitalization. We have organized the manuscript in a manner compliant with the STROBE (Strengthening the Reporting of Observational Studies in Epidemiology) statement.

**Results:**

Acute kidney injury occurred within the two groups (*p* = 0.007 and *p* = 0.001, respectively). The early-morning blood glucose levels (149.7±44.0 mg/dL) and average blood glucose levels (185.6±52.0 mg/dL) were better in Group A (*p* = 0.01, *p* = 0.02). Group A patients also had lower glucose variability than Group B at the different time points (*p*<0.05). Group A also had earlier renal recovery. More relevant pathogens were identified from blood in Group B (*p* = 0.038).

**Conclusions:**

Early-morning fasting and mean blood glucose levels and their variability can be good indicators of severe infection and predictors of renal outcome in type 2 diabetic patients with CKD and UTI.

## Introduction

Urinary tract infection (UTI) is a common disease in type 1 and 2 diabetic patients, and diabetic patients are prone to UTI as well [1]. Among diabetic patients, 9–20% of women and 3–11% of men may develop UTI [2], which may result in longer hospitalizations (3- to 5-fold) [3] and higher mortality (7.6% versus 1.6%) [4] in patients with diabetes than in those without diabetes. Additionally, in a previous study, we found that UTI can also lead to acute kidney injury (AKI) in type 2 diabetic chronic kidney disease (CKD) patients who had an estimated glomerular filtration rate (eGFR) of <30 mL/min on admission [5]. Furthermore, AKI episodes can be independent risk factors for renal progression in diabetes CKD patients [6]. The RIFLE criteria for AKI were defined according to the Acute Dialysis Quality Initiative consensus in 2002, and denoted that GFR can abruptly decrease by more than 25% within 1–7 days after admission and may be sustained for longer than 1 day. For almost all type 2 diabetic patients with advanced CKD, renal dysfunction did gradually revert within 6 months after the UTI if they survived and were free from long-term dialysis [5]. The rate of renal recovery was also essential; a shorter recovery period would improve health and reduce the risk of another episode of infection. Possible factors that could affect renal recovery included the baseline GFR, severity or duration of sepsis, and concurrent co-morbidities. Critically ill patients with lower glucose variability have lower short-term or hospital mortality than patients with higher glucose variability [7]. However, the impact of glucose and its fluctuations on renal function during hospitalization from nonfatal infection has not been reported. This study aimed to clarify the association of glucose and its variability during admission and renal outcome in type 2 diabetic patients with CKD and episodic UTI.

## Materials and Methods

**Figure 1:**
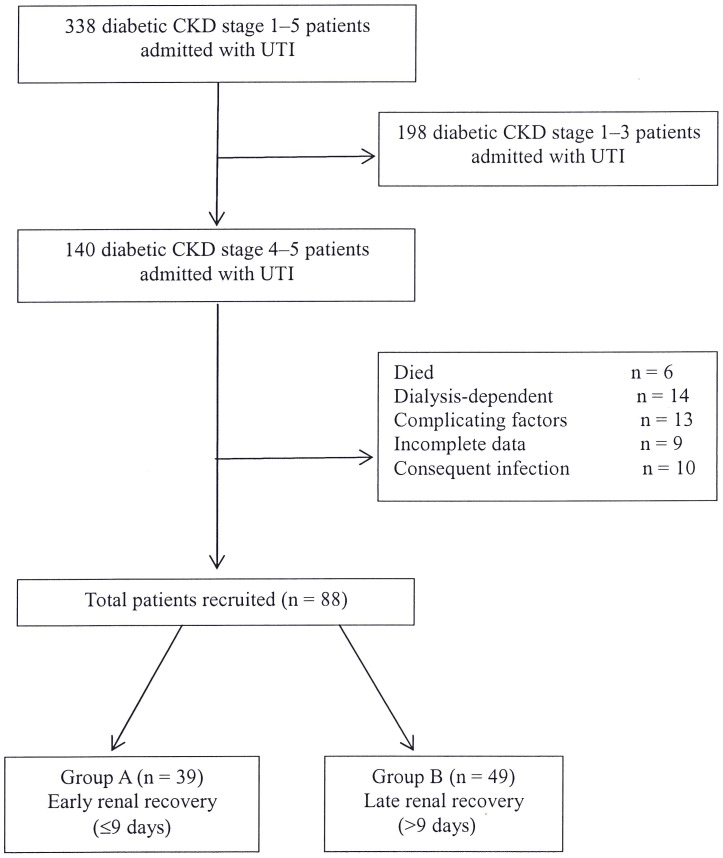
Participant flow diagram depicting the screening/enrollment process.

We enrolled patients that had been managed under CKD care programs, who also had UTI and were admitted to our hospital from 2001 to November 2013. The admission criteria for UTI in our hospital included the presence of a systemic inflammatory response syndrome or a complicated condition involving a major organ. We excluded patients who had untreated obstructive uropathy, those who died, those who were dialysis-dependent after infection, or those who had subsequent hospital-acquired infections. To improve the quality of reporting in observational studies, the manuscript was organized in a manner compliant with the STROBE (Strengthening the Reporting of Observational Studies in Epidemiology) statement [8]. The patient flow chart is shown in Figure 1, which includes the number of patients recruited and excluded from the study. The Modification of the Diet in Renal Disease (MDRD) formula was applied for GFR estimation. The formula used for eGFR was the following:
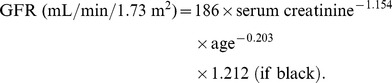



AKI was defined using the RIFLE criteria. When the decrease in eGFR returned to <10% of baseline renal function, the patient was determined to be in renal recovery as defined in previously published manuscripts [9], [10]. Based on the date of renal recovery (GFR decrease within 1–7 days and that was sustained for longer than 1 day) after admission, we divided these patients into early- (≤9 days, Group A) and late-recovery (>9 days, Group B) groups. The basic characteristics of these patients were collected and analyzed in Table 1. The continuous and categorical variable differences, including blood glucose (Table 2–4) and bacteriology (Table 5), of the two groups were assayed separately by the Student's t-test, the Mann–Whitney U test, and the chi-squared test. The mean glucose level and its variability (calculated by the standard deviation and the coefficient of standard deviation: SD/mean) at four fixed time points (fasting, midday pre-meal, evening pre-meal, evening post-meal) during the hospitalization were compared. The patients' survival rates after episodic UTI were analyzed using the Kaplan–Meier method.

**Table 1:** Patient characteristics.

	Group A (n = 39)		Group B (n = 49)		
	Mean	SD	Mean	SD	*p*
Age‡	67.8	8.7	68.4	14.1	0.80
Gender, male†	26.3%		26.5%		0.98
Hypertension†	76.3%		63.3%		0.009*
CVD†	23.7%		24.5%		0.86
Hyperlipidemia†	18.4%		16.3%		0.61
Liver†	15.8%		16.3%		0.89
Stroke†	21.1%		22.5%		0.75
SBP (mmHg)‡	135	26	121	17	0.057
DBP (mmHg)‡	73	17	72	13	0.78
eGFR (ml/min)‡	16.9	7.9	15.4	7.7	0.47
BUN (mg/dL)‡	61.4	41.7	59.6	33.6	0.84
Cr (mg/dL)‡	4.09	2.9	3.99	2.3	0.86
ALT (U/L)‡	23.3	12.3	34.6	29.6	0.03*
HbA1C (%)‡	7.7	2.0	8.2	2.8	0.30
Cholesterol (mg/dL)‡	150.8	62.1	169.6	60	0.79
Triglyceride (mg/dL)‡	170.3	77.9	209.5	80.1	0.51
HDL (mg/dL)‡	37.7	12.8	39.4	17.7	0.90
LDL (mg/dL)‡	89.8	35.6	102.9	44.8	0.76
Uric acid (mg/dL)‡	6.3	1.6	7.9	2.2	0.08
Albumin (g/dL)‡	2.8	0.7	2.7	0.6	0.37
Na (mEq/dL)‡	132.3	6.8	133.0	7.0	0.61
K (mEq/dL)‡	4.6	1.0	4.2	1.1	0.15
WBC (×10^3^)‡	11.3	5.5	13.5	8.3	0.10
Hemoglobin (g/dL)‡	10.4	2.4	10.2	2.5	0.72
Hematocrit (%)‡	30.5	7.7	30.0	7.4	0.77
UPCR (g/g)‡	1.8	2.0	1.1	1.2	0.31
Hospital days§	6.78	3.68	11.5	10.8	0.019*
(median)	6.0		7.5		

Abbreviations: CVD, cardiovascular disease; ALT, alanine aminotransferase; WBC, white blood cell; UPCR, urine protein–creatinine ratio; SBP, systolic blood pressure; DBP, diastolic blood pressure.

† chi-square test.

‡ student's t-test.

§ Mann–Whitney U test.

*p<0.05.

**Table 2:** Blood glucose levels and renal outcome.

	Group A		Group B		
Glucose (mg/dL)	mean	SD	mean	SD	*p*
Fasting	149.7	44.0	181.2	64.5	0.01*
Midday pre-meal	203.7	60.8	228.5	81.9	0.17
Evening pre-meal	209.9	62.5	234.6	74.4	0.11
Evening post-meal	216.0	54.5	228.2	68.8	0.41
mean	185.6	52.0	216.7	69.5	0.02*

Abbreviation: SD, standard deviation.

*p<0.05.

**Table 3:** Glucose variability: standard deviation (SD) and renal outcome.

	Group A		Group B		
Glucose (mg/dL)	SD mean	SD	SD mean	SD	*p*
Fasting	46.6	37.9	64.0	32.6	0.03*
Midday pre-meal	64.6	40.1	72.1	40.2	0.46
Evening pre-meal	66.1	37.4	84.6	40.5	0.04*
Evening post-meal	64.0	37.7	83.0	38.3	0.04*

*p<0.05.

**Table 4:** Glucose variability: coefficient of variation (CV) and renal outcome.

	Group A		Group B		
Glucose (mg/dL)	CV mean	SD	CV mean	SD	*p*
Fasting	0.29	0.19	0.36	0.14	0.001*
Midday pre-meal	0.30	0.15	0.32	0.16	0.03*
Evening pre-meal	0.32	0.18	0.36	0.15	0.13
Evening post-meal	0.28	0.13	0.35	0.15	0.02*

*p<0.05.

**Table 5:** Bacteriology.

	Group A	Group B	*p*
Blood culture	7	17	0.038*
GNB	5	15	
GPC	2	2	
Urine culture	34	39	0.18
GNB	21	30	
GPC	5	6	
fungus	5	2	
others	3	1	

Abbreviations: GNB, gram-negative bacillus; GPC, gram-positive cocci.

chi-square test.

*p<0.05.

### Ethics Statement

The CKD care program including ethics and patients' right was guided by the Clinical Care Program Certification and Joint Commission International. This protocol was also approved by the Institutional Review Board of the Changhua Christian Hospital, Taiwan. All subjects gave written informed consent to participate. If patients could not be contacted in any way, the data were categorized as incomplete.

## Results

**Figure 2:**
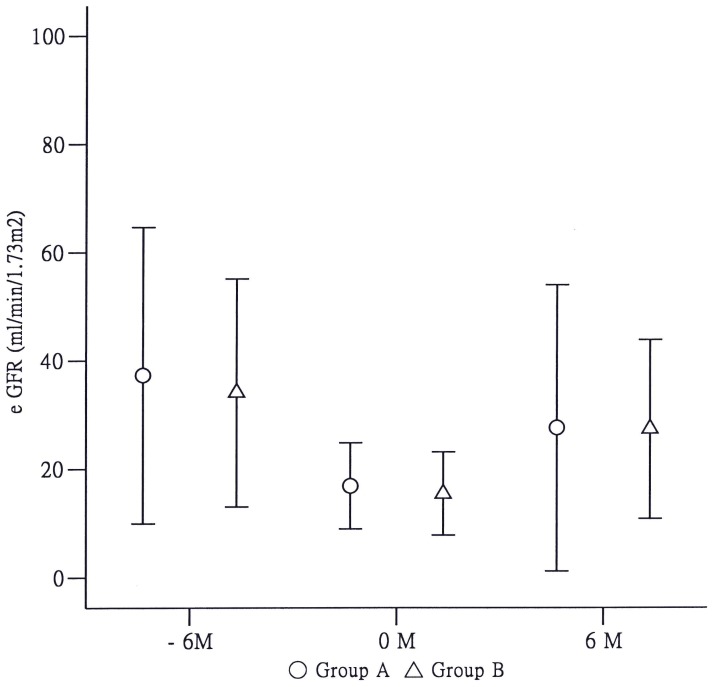
Changes in estimated glomerular filtration rate (eGFR) over time within the early- and late-recovery groups. T (0) represents the time point on admission from urinary tract infection; the “plus” and “minus” symbols represent the time interval after and before admission, respectively. Acute kidney injury occurred in diabetic patients with chronic kidney disease [eGFR at T (0) versus T (−6); *p* = 0.007 in Group A; *p* = 0.001 in Group B], and eGFR reverted to the trend 6 months later if urinary tract infection was cured.

One hundred and forty diabetic CKD patients with eGFR of <30 mL/min who were admitted to the hospital with UTI during the 13 years were evaluated for the study. Fifty-two patients had been excluded because of various causes as reported in Figure 1. Eighty-eight patients (65 females, 23 males) were included. Thirty-nine patients had recovered renal function early (≤9 days, Group A), and 49 patients recovered function late (>9 days, Group B). The baseline characteristics of the patients are listed in Table 1. The age and incidence of comorbidities, such as cardiovascular disease, stroke, and liver disease, were compared between the two groups. The biochemical and hematological data were balanced in both groups, with the exception of alanine aminotransferase (ALT). The estimated GFR series are illustrated in Figure 2. AKI occurred during admission in the two groups (*p* = 0.007 and *p* = 0.001, respectively), and almost all diabetic patients gradually recovered within 6 months. In contrast, 34 non-diabetic CKD stage 4–5 patients (n = 544; 0.063%, data not shown) were admitted for urosepsis during this period. No significant AKI was observed (*p* = 0.39, data not shown).

Microorganisms detected in blood were more notable in Group B (Table 5; *p* = 0.038). Urine pathogen culture rates found between both groups were comparable. E. coli was the most commonly isolated pathogen. Moreover, approximately 32% (12 in Group A; 16 in Group B) of patients had cystitis within 3 months before the urosepsis episodes. Two-week antibiotic regimens were prescribed at least for patients with complicated UTI, including first- and second-generation cephalosporins and third-generation cephalosporin/or quinolone (33, 6 in group A; 38, 11 in Group B, respectively). Aminoglycosides were avoided owing to their nephrotoxicity.

The mean early-morning blood glucose levels (149.7±44.0 mg/dL) and average blood glucose levels (185.6±52.0 mg/dL) were better in Group A (*p* = 0.01, *p* = 0.02; Table 2). Patients in Group A also had lower glucose variability based on the differences in the standard deviation and the coefficient of variation (SD/mean) than patients in Group B at the different time points. There was more relevance in the fasting and the evening post-meal blood glucose levels (Table 3 and 4; *p*<0.05). The trend of glucose variability was also consistently observed at the midday pre-meal and evening pre-meal time points.

The two groups had equivalent long-term patient survival (data not shown). We also adjusted for possible confounding factors that may have contributed to renal recovery using multivariate cox proportional hazard regression, including age, severity of infection, and glucose and blood pressure control (Table 6).

**Table 6:** Cox proportional hazard regression: hazard ratio of variables and renal injury.

Variable	HR	95% CI	*p*
Glucose (mean)	1.006	1.002–1.010	0.003*
Age	0.979	0.951–1.008	0.150
Hypertension	0.329	0.174–0.619	0.001*
WBC	0.955	0.889–1.025	0.202
HbA1C	0.957	0.844–1.086	0.498

*p<0.05.

## Discussion

Diabetes and its complications are the leading cause of end-stage renal disease worldwide. Diabetes patients are vulnerable to UTI. Further, UTI may cause AKI in diabetic CKD patients with an eGFR of <30 mL/min. Furthermore, repeated episodes of AKI are related to rapid progression of renal-failure. The main finding of this study was that better blood glucose control and lower glucose variability during hospitalization in type 2 diabetic patients with CKD could predict earlier renal recovery from UTI. Because these patients were not critically ill and remained in the ward, glucose measurement at four different time points (fasting, midday pre-meal, evening pre-meal, evening post-meal) was applicable, instead of continued glucose monitoring.

High glycosylated hemoglobin in the diabetic population has been associated with a high incidence of microangiopathy, macroangiopathy, and mortality [11]. Long-term glucose variability has also played a role in the development of microangiopathy and macroangiopathy [12], [13]. A study by Monnier et al even suggested that glucose variability may be more deleterious than chronic hyperglycemia in the development of diabetic complications [14]. Acute blood glucose fluctuations during hospitalization for various critical conditions have predicted short-term or in-hospital mortality [15]. The better outcomes were obtained in critical patients with lower glucose variability. This scenario was also found in non-diabetic critically ill patients [16]. Jeon et al demonstrated that pre- and post-operative glucose levels and their variability were associated with the risk of surgical site infection or in-hospital death [17]. Additionally, increased blood glucose variability during hospitalization has been a precursor of sepsis and mortality in burn patients as reported by Alexander et al [18]. Furthermore, a meta-analysis by Haga et al revealed that tight glycemic control might reduce early mortality, the incidence of atrial fibrillation, and the time spent in the intensive care unit after cardiac surgery [19].

In contrast, in data from the reanalysis of the HEART2D study, Siegelaar et al found that a decrease in glucose variability did not reduce cardiovascular events in type 2 diabetic patients after an acute myocardial infarction [20]. The tight blood glucose control in critical patients does not reduce mortality but does increase the risk of hypoglycemia according to meta-analysis studies [21], [22]. In the NICE-SUGAR Study, a blood glucose target of 180 mg/dL or less resulted in lower mortality than did a target of 81–108 mg/dL [23]. For non-critically-ill patients treated with insulin, according to the Standards of Medical Care in Diabetes 2013, the pre-meal glucose target should be around 140 mg/dL and post-meal glucose target should be around 180 mg/dL [24]. However, because advanced CKD diabetic patients can be at higher risk of hypoglycemia, sugar control that is less tight seems reasonable. Nevertheless, no current guidelines have been recommended for these patients.

Several possible mechanisms may account for chronic glucose variability. Short-term Type 1 diabetes mellitus (DM) participants with residual β-cell function had lower glucose variability than longer-term Type 1 DM participants (27% versus 42%, respectively; *p*<0.001) [25]. An autonomic imbalance from increased hypothalamic-pituitary axis activity and cortisol levels can also lead to worsening glucose variability and neuropathy [26], [27]. The study by Monnier et al indicated that acute glucose excursion triggered more oxidative stress than chronic sustained hyperglycemia in type 2 diabetic patients. There is a linear correlation between 24-hour urinary excretion rates of 8-Iso Prostaglandin F2 and glycemic variability [28]. The antidiabetic agents, dipeptidyl peptidase IV inhibitors, were found to reduce glucose fluctuations and plasma nitrotyrosine, IL-6, and IL-18 levels (p<0.05) in type 2 diabetic patients after 3 months of treatment [29]. Additionally, acute glucose elevation can be highly predictive of infection in critically injured trauma patients, as reported by Bochicchio et al [30]. In our study, it was evident that increased glucose variability in type 2 diabetic patients with CKD and eGFR of <30 mL/min could have delayed the renal recovery from UTI. Additionally, those who had delayed renal recovery also had longer hospital stays.

Another interesting finding was the remarkable pathogen identification rate from blood in Group B. Both C-reactive protein and procalcitonin concentrations can suggest bacterial infection in emergency patients [31], [32]. This may imply that hospitalized patients with higher blood glucose and greater variability were in course of more severe inflammation and oxidative stress. Perhaps, infections with concurrent glucose variability may simultaneously influence renal injury and recovery. Elevated liver enzymes were frequently found in septic conditions. Several hepatic biomarkers, including bilirubin, albumin, alkaline phosphatase, aspartate, ALTs, and lactate dehydrogenase, had been evaluated in association with the mortality rate in the intensive care unit by Marshall et al. However, no ideal biomarker has been identified at this time [33].

Several methods were applied for measuring the glucose variability [34]. However, from previous systematic reviews and our study, the magnitude of glycemic variability was highly correlated with the level of the mean glucose value. The degree of correlation of the different measures of glycemic variability was also high [35].

Some limitations in our current study included the following: (1) relative small sample size; (2) single-center evaluation; (3) retrospective analysis; and (4) the use of monitored glucose at fixed time points, instead of continuous glucose monitoring. Whether aggressive control of blood glucose levels during ward hospitalization could improve the recovery of the renal dysfunction would need to be evaluated in a controlled study in the future. However, at least, the glucose levels are a good indicator of severe infection, organ dysfunction, and renal outcome in type 2 diabetic patients with CKD and UTI.

## Conclusions

Early-morning fasting and mean blood glucose levels and glucose variability during hospitalization are good indicators of severe infection, and also predictors of renal outcome in type 2 diabetic patients with CKD and UTI.
